# A Case of Canine Hepatitis with Hepatocellular Attack by Non-Neoplastic Perforin-Laden Lymphocytes

**DOI:** 10.3390/vetsci12030211

**Published:** 2025-03-01

**Authors:** Shimon Furusato, Eriko Kondo, Ikki Mitsui, Yu Tsuyama

**Affiliations:** 1 Shinagawa WAF Animal Hospital, Shinagawa 141-0032, Japan; shimon.furusato@gmail.com (S.F.); kondo_e@icloud.com (E.K.); 2 Laboratory of Veterinary Anatomy, Faculty of Veterinary Medicine, Okayama University of Science, Imabari 794-8555, Japan; mitsui@no-boundaries.jp

**Keywords:** autoimmune hepatitis, canine chronic hepatitis, hepatocellular apoptosis, immunohistochemistry, immunosuppressive therapy, perforin-containing T lymphocytes

## Abstract

The etiology of canine chronic hepatitis (CH) is unknown, although an autoimmune background has been suggested in some cases of canine CH. An 11 y old spayed female Norwich Terrier showed a marked elevation of liver enzymes with hyperbilirubinemia, regenerative anemia, and thrombocytopenia. A bacterial culture of the surgically excised liver tissue and bile was negative. The histological features of the liver biopsy resembled those of human autoimmune hepatitis except for a paucity of intralesional plasma cells. It was established through immunohistochemistry that CD3-positive perforin-containing T lymphocytes had actively infiltrated the patient’s liver causing hepatocellular apoptosis, implying an autoimmune attack on hepatocytes. The patient’s general condition improved, with normalization of platelet and serum total bilirubin levels, after immunosuppressive therapy with prednisolone and cyclosporine, whereas liver enzymes did not reach the reference interval. The dog died 11 months after the initiation of immunosuppressive therapy. These pathological findings may be one aspect of autoimmune mediation in canine CH.

## Simple Summary

The cause of canine chronic hepatitis (CH) remains unknown, but an autoimmune basis is suspected in some cases. An 11-year-old spayed female Norwich Terrier presented with elevated liver enzymes, hyperbilirubinemia, regenerative anemia, and thrombocytopenia. Bacterial cultures of liver tissue and bile were negative. Liver histology resembled human autoimmune hepatitis, except for a lack of plasma cells. Immunohistochemistry revealed the infiltration of CD3-positive, perforin-containing T lymphocytes, causing hepatocellular apoptosis, suggesting an autoimmune attack. Treatment with prednisolone and cyclosporine improved the dog’s overall condition, normalizing platelet and total bilirubin levels, though liver enzymes remained elevated. The dog died 11 months after starting treatment. These findings suggest that an autoimmune mechanism may contribute to canine CH.

## 1. Introduction

Canine chronic hepatitis (CH) is pathologically characterized by hepatocellular apoptosis or necrosis, variable mononuclear or mixed inflammatory infiltrates, hepatocellular regeneration, and fibrosis [1]. Although various etiologies have been proposed for canine CH, including infectious, metabolic, toxic, and immune, most cases were categorized as idiopathic due to the lack of sound evidence to support a specific cause [2,3]. Immunosuppressants, particularly corticosteroids, have been used to control idiopathic CH or breed-specific hepatitis, showing partial improvements [4,5,6], suggesting the possible role of immune-mediated pathogenesis underlying CH. Indeed, an autoimmune background has been considered in some canine CH cases on the basis of an elevated titer of serum autoantibodies [3]. However, there is currently a lack of information on the pathogenesis of immune-mediated hepatitis in dogs and no established criteria or optimal treatment [7]. Understanding the mechanisms behind this immune-mediated attack on hepatocytes is essential for developing effective treatments and improving the prognosis for affected dogs. Here, we present pathological findings that suggest immune mediation in a dog with CH.

## 2. Case Description

**Figure 1:**
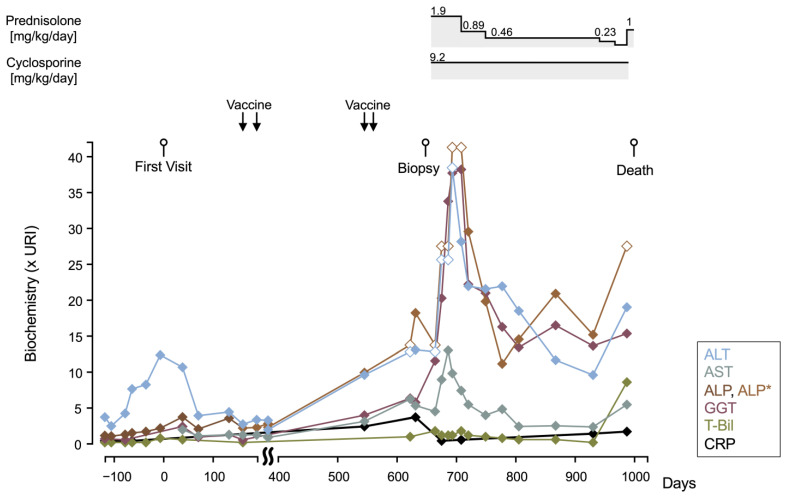
Chronological change in blood biochemistry values. The timings of vaccination and immunosuppressant (prednisolone and cyclosporine) administration are also shown. Biochemical data were converted to multiples of the upper RI (× URI). * RI varied due to a change in the methodology. The values indicated by white diamonds represent measurements exceeding the detection limit. Alanine aminotransferase, ALT; aspartate transaminase, AST; alkaline phosphatase, ALP; gamma-glutamyl transpeptidase, GGT; serum total bilirubin, T-Bil; C-reactive protein, CRP.

An 11 y old spayed female Norwich Terrier weighing 6 kg presented (day 0) with a history of subclinical elevation of liver enzymes for several years. The dog was up to date with rabies and core vaccines against canine adenovirus 1 and 2, parvovirus, distemper, and parainfluenza virus. Vaccines against *Leptospira canicola* and *L. icterohaemorrhagiae* had been annually administered. No abnormal findings, including liver dysfunction or imaging features, were observed, except for subjective microhepatica. There was no history of exposure to hepatotoxic drugs, such as phenobarbital, primidone, phenytoin, lomustine, carprofen, oxibendazole, amiodarone, aflatoxin, or cycasin [3]. The chronological change in the serum biochemistry as multiples of the upper reference interval (× URI) and clinical summaries are shown in Figure 1. On day 623, hepatobiliary parameters were markedly elevated (alanine aminotransferase > 12× URI, RI 17–78 U/L; aspartate transaminase 6.3× URI, RI 17–44 U/L; alkaline phosphatase > 13× URI, RI 0–89 U/L; gamma-glutamyl transpeptidase 6.4× URI, RI 0–14 U/L; serum total bilirubin 1.2× URI, RI 0–0.5 mg/dL) with regenerative anemia (PCV 32.8%, RI 37.3–61.7%) and thrombocytopenia (platelet 53,000 cells/μL RI 148,000–484,000 cells/μL). Coagulation assays indicated a hypercoagulable state and hyperfibrinolysis (thrombin–antithrombin complex 1.0 ng/mL RI 0–0.2 ng/mL D-dimer 9.07 μg/mL RI 0–2 μg/mL FUJIFILM VET Systems, Tokyo, Japan). The patient also experienced weight loss, intermittent vomiting, and anorexia.

**Figure 2:**
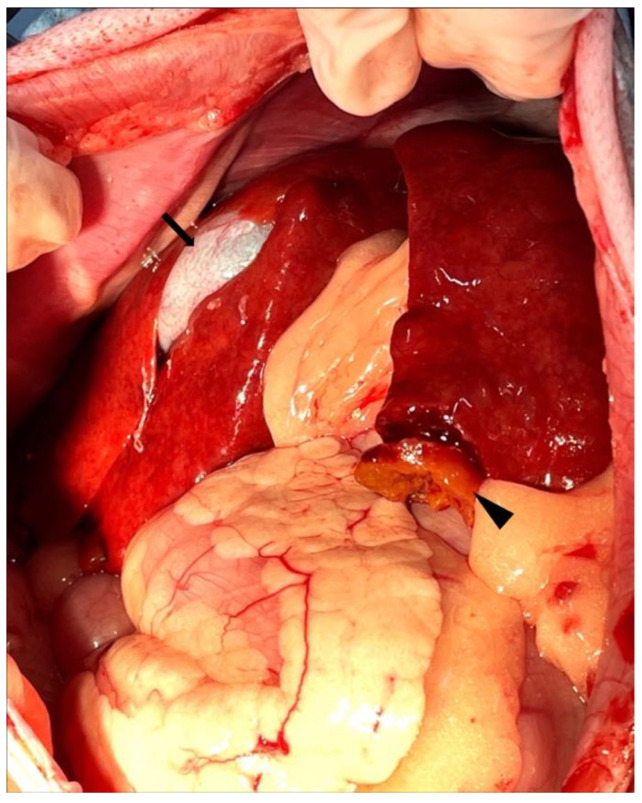
Gross appearance of the liver. The liver lobes have rounded edges, multiple nodules, and a rough capsular surface. The tip of the left lateral liver lobe was ligated for biopsy (arrowhead). The gallbladder was slightly dilated (arrow).

**Figure 3:**
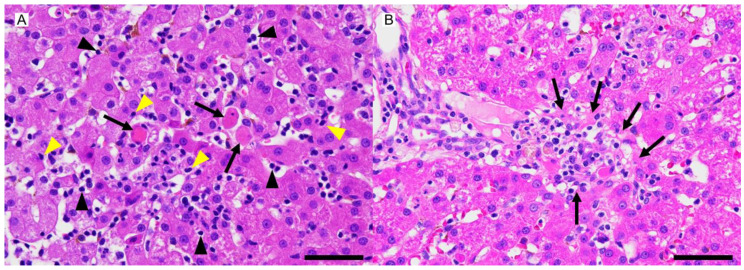
Histopathological findings from a dog with suspected immune-mediated hepatitis. (**A**): Individual necrosis (apoptosis) of hepatocytes (arrows), sinusoidal infiltration of small lymphocytes (black arrowheads), disruption of hepatic cords by small lymphocytes (emperipolesis, yellow arrowheads), and irregularly aligned hepatic cords are present. H&E stain. Bar = 50 μm. (**B**): The limiting plate is disrupted by lymphocytic infiltration and hepatocellular necrosis/apoptosis (interface hepatitis, arrows). H&E stain. Bar = 50 μm.

**Figure 4:**
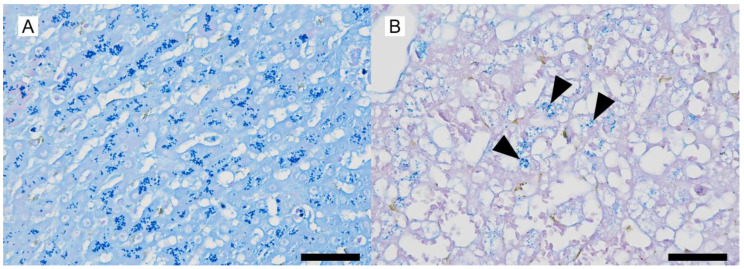
Histochemical findings for a dog with suspected immune-mediated hepatitis. (**A**): Numerous (Thornburg grade 5) intracytoplasmic copper granules are present in the hepatocytes of the control dog afflicted with copper-storage hepatopathy. Victoria blue method. Bar = 50 μm. (**B**): Small numbers (Thornburg grade 1) of intracytoplasmic copper granules are present in the hepatocytes (arrowheads) of the index case. Victoria blue method. Bar = 50 μm.

**Figure 5:**
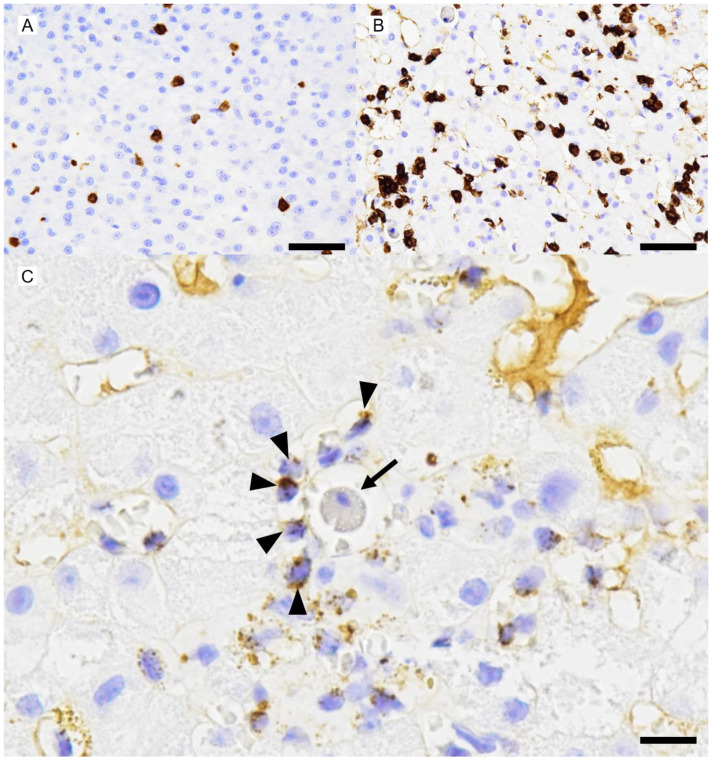
Immunohistochemical (IHC) findings for a dog with suspected immune-mediated hepatitis. (**A**): CD3-immunopositive lymphocytes are sparse in the liver of the control healthy dog. IHC. Bar = 50 μm. (**B**): CD3-immunopositive lymphocytes are numerous in the liver of the index dog. IHC. Bar = 50 μm. (**C**): Lymphocytes containing perforin-immunoreactive granules (arrowheads) in the cytoplasm surround an apoptotic hepatocyte (arrow). IHC. Bar = 25 μm.

A wedge liver biopsy and bile sampling were conducted during exploratory laparotomy on day 648. The liver lobes were subjectively judged to be smaller than normal with rounded edges and a rough capsular surface (Figure 2). Nodules ranging from 1 to 2.2 mm in diameter were noted in the parenchyma. Bile was normal in color and consistency and contained no sediment. The biopsy was fixed in 10% neutral-buffered formalin, processed routinely, embedded in paraffin wax, sectioned at 4 µm thickness, and stained with H&E. A histopathological examination of the liver sample was performed by a board-certified veterinary anatomic pathologist (Mitsui). Microscopically, the hepatic cords were 1–3 cells thick and haphazardly arranged (Figure 3), in contrast to their normal 1–2-cell-thick straight arrangement. The hepatocytes were mildly to moderately swollen with multiple small (1–2 µm diameter) intracytoplasmic pale eosinophilic vacuoles without nuclear displacement (hepatocellular vacuolar degeneration). Apoptosis/death of scattered individual hepatocytes with dropout from the hepatic cords was observed (Figure 3). Mitotic figures, indicative of regenerative attempts, were uncommon. Sinusoids were diffusely congested with an increased number of small lymphocytes (Figure 3). These lymphocytes lacked nuclear atypia, pleomorphism, or mitotic figures but had a slightly increased amount of clear cytoplasm. These lymphocytes also infiltrated the hepatic cords (Figure 3). Few plasma cells were admixed with the lymphocytes (Figure 3). Scattered sinusoidal aggregates of macrophages, laden with fine amber granules (likely lipofuscin) or coarse brown granules (likely hemosiderin), were present. Some portal tracts were mildly expanded by fibrosis and/or a mild infiltration of small lymphocytes, plasma cells, neutrophils, and macrophages, with lymphocytes being predominant. The limiting plate in some lobules was disrupted by lymphocytic infiltration and hepatocellular necrosis/apoptosis (interface hepatitis, Figure 3). There were no lesions in the interlobular bile ductules, arteries, or veins. Multiple regenerative nodules composed of 2–4-cell-thick hepatic cords were also present. These regenerative nodules had less severe sinusoidal congestion and fewer small lymphocytes than the surrounding parenchyma. Lymphatics around the central veins and portal tracts were moderately to markedly dilated. The hepatic capsule was unremarkable. Small numbers (equivalent to Thornburg grade 1) [8] of isolated hepatocellular intracytoplasmic copper granules were detected in the hepatic lobules in the sections stained by the Victoria blue method (Figure 4). Equivalent results were obtained using the rhodanine stain. In addition, Masson trichrome staining revealed a slightly increased amount of collagen in the space of Disse throughout the specimen. No neoplastic cells, bacterial/fungal agents, viral inclusions, bile plugs, or portal/periportal/bridging fibrosis were observed in the examined sections. Of note was the lack of granulomatous hepatitis, a feature of hepatic leptospiral infections [9]. Immunohistochemistry was performed using the antibodies listed in Table 1. Intralesional CD3-positive lymphocytes were more numerous than in the liver of a healthy age-matched dog (Figure 5). These CD3-positive lymphocytes had a few fine-to-coarse intracytoplasmic granules that were immunopositive for perforin and rarely surrounded apoptotic hepatocytes (Figure 5). The liver specimen from the age-matched healthy dog did not contain perforin-laden lymphocytes. The CD3-positive perforin-containing lymphocytes seldom infiltrated the hepatocellular cytoplasm (emperipolesis; Figure 3). CD20-positive lymphocytes were seldom present in sinusoids. The number of MUM1-positive (nuclear stain) plasma cells was low and they tended to be confined to the portal tracts and periportal areas. The numbers of hepatic stellate cells (HSCs) in 10 high-power fields (2.37 mm^2^, using an FN22 ocular lens) were manually counted on specimens immunostained with anti-αSMA antibody in accordance with the published literature [10,11]. The patient’s specimen had 100 HSCs, whereas the control specimen had 38 HSCs in the same observation field of 2.37 mm^2^.

These histological features are consistent with those of CH based on the criteria proposed by the World Small Animal Veterinary Association (WSAVA) Liver Standardization Group [1]. The negative bacterial culture of the liver and the bile and the low-grade accumulation of copper in the hepatocytes ruled out bacterial- or copper-associated hepatitis in this case. Most importantly, the infiltration of perforin-laden CD3-positive lymphocytes suggested an autoimmune attack against the dog’s hepatocytes.

We started immunosuppressive therapy of prednisolone (1.9 mg/kg q24h) and cyclosporine (4.6 mg/kg q12h) concurrent with famotidine (0.93 mg/kg q24h) on day 657 (Figure 1). Although the liver enzyme levels did not normalize, the patient’s general condition improved, with the normalization of platelet, serum total bilirubin, and C-reactive protein levels 18 days after immunosuppressive therapy initiation, which was maintained for 10 months. Prednisolone was tapered from day 708, and the liver enzyme levels tended to decrease and the patient’s general condition remained stable. To treat pyoderma, prednisolone was discontinued and cephalexin was prescribed on day 967, but vomiting, diarrhea, and a decreased appetite occurred a few days later. The antibiotic was changed to orbifloxacin but vomiting continued. Twenty days later, the dog’s general condition worsened with a marked elevation of liver enzymes, hyperbilirubinemia, mild non-regenerative anemia, and obvious liver atrophy on ultrasonography. Immunosuppressive therapy was intensified by increasing the prednisolone dose to 1 mg/kg q24h, and cyclosporine was switched to mycophenolate mofetil (15.8 mg/kg q12h). The dog’s condition continued to deteriorate, and it died at home on day 999. No autopsy was performed.

## 3. Discussion

The histologic findings in this case are consistent with descriptions for canine CH, in which inflammation typically originates in portal areas and spreads into adjacent hepatic parenchyma with the destruction of the limiting plate (interface hepatitis) [3,12]. Other histologic features of canine CH include bridging necrosis between portal triads or between portal areas and central veins, bile duct degeneration, multifocal necrosis, hepatocellular regeneration, and collagen deposition in the space of Disse [12]. In addition, abnormally high hepatocellular copper accumulation is an important feature of CH in some breeds, including the West Highland white terrier, Bedlington terrier, and Dobermann pinscher [12,13]. On the other hand, no significant hepatocellular copper accumulation was detected in cohorts of English springer spaniels, despite the presence of hepatocellular necrosis/apoptosis, lymphoplasmacytic inflammation, interface hepatitis, and variable fibrosis [4,14].

The present case also shared many of the histologic findings described for human autoimmune hepatitis (AIH). Confirmation of human AIH is based on a complex algorithm that includes abnormal levels of serum globulins or IgG, characteristic circulating autoantibodies (antinuclear antibodies, smooth muscle antibodies, or antibodies to liver kidney microsome type 1), and the exclusion of viral involvement, ruling out excessive alcohol intake or toxic drug administration, typical hepatic histopathological features, female predisposition, and responsiveness to immunosuppression [15,16]. Typical but not pathognomonic histologic features of human AIH are lymphoplasmacytic inflammation that can include plasma cell clusters; emperipolesis (intact lymphocytes within hepatocytes); hepatocellular rosettes; interface hepatitis; portal-based fibrosis; and the exclusion of other liver diseases [16]. Criteria for the diagnosis of canine immune-mediated hepatitis have not been established [3]. Since the histologic criteria for human AIH seem to be undergoing a transition, influenced by an ongoing debate on the inclusion of emperipolesis and hepatocellular rosettes [16,17], it would be prudent to avoid rigorous comparisons between human AIH and the present canine case from a purely histologic standpoint. However, the only obvious histologic difference was fewer plasma cells in the hepatitis lesions of this dog than in typical cases of human AIH [16].

Phenotyping of the infiltrating lymphocytes in human AIH has been limited, with past investigations focused on regulatory T cells [18,19]. According to the veterinary literature, the liver has resident natural killer T lymphocytes (so-called pit cells) [12]. The exact source of the T lymphocytes with cytoplasmic perforin granules in the liver of the present case remains unclear, but they may be pit cells or circulating lymphocytes sensitized to hepatocellular antigens. The possibility that they were neoplastic lymphocytes was ruled out by their small number, the reduced liver size, a lack of remarkable sinusoidal widening [20], and evidence of their attack on hepatocytes, as demonstrated by the single-cell necrosis/apoptosis.

Evaluation of the degree of fibrosis is important for staging in the liver pathology [12]. In the present case, there was a slight increase in collagen in the space of Disse throughout the specimen, as depicted by the Masson trichrome stain. We did not have a reliable histomorphological scale to evaluate the magnitude of this increase, but the lesion appeared to still be in the early stage of the disease course, as judged by a lack of portal, periportal, or centrilobular fibrosis. Immunohistochemistry using anti-αSMA antibody would have been feasible, because it would have allowed us to perform numerical evaluation rather than qualitative assessment. An evaluation of hepatic fibrosis using special stain and/or immunohistochemistry will be necessary in future investigation to precisely date the lesion and correctly predict the disease outcome.

In this case, immunosuppressive therapy failed to achieve complete biochemical remission despite maintaining a good quality of life for 10 months. The failure to respond to immunosuppressive therapy, however, does not exclude an immune-mediated pathogenesis. Previous studies reported that remission was not achieved with cyclosporine in 21% of dogs with idiopathic CH or with predniso(lo)ne in 58% of humans with AIH [6,21]. The novelty of this case report is the infiltration of CD3-positive perforin-containing T lymphocytes in the hepatic regions, which suggests an autoimmune attack on hepatocytes.

## 4. Conclusions

We reported a case of canine CH that shared many histologic features with human AIH, except for a paucity of intralesional plasma cells. Immunohistochemistry further revealed active infiltration of CD3-positive perforin-containing T lymphocytes in the patient’s liver, suggesting an autoimmune attack on hepatocytes. These pathological findings may represent one aspect of autoimmune mediation in canine CH.

## Figures and Tables

**Table 1:** Primary antibodies used in immunohistochemistry.

Antibody To	Host	Type, Clone	Dilution	Source	Catalog Number
CD3	Mouse	Monoclonal, LN10	Ready to use	Leica Biosystems	PA0553
CD20	Mouse	Monoclonal, L26	×200	Leica Biosystems	NCL-L-CD20-L26
Perforin	Mouse	Monoclonal, 5B10	Ready to use	Abcam	ab75573
MUM1	Mouse	Monoclonal, MUM1p	×100	Dako	M7259
αSMA	Mouse	Monoclonal, asm-1	Ready to use	Leica Biosystems	PA0943

## Data Availability

The data presented in this study, which includes patient personal information, are available in an anonymized format upon request from the corresponding author.
